# The role of surgical resection in primary central nervous system lymphoma: a single-center retrospective analysis of 70 patients

**DOI:** 10.1186/s12883-021-02227-3

**Published:** 2021-05-11

**Authors:** Shiqiang Wu, Junwen Wang, Weihua Liu, Feng Hu, Kai Zhao, Wei Jiang, Ting Lei, Kai Shu

**Affiliations:** Department of Neurosurgery, Tongji hospital, Tongji Medical College, Huazhong University of Science and Technology, 1095# Jiefang Avenue, Wuhan, 430030 Hubei China

**Keywords:** Primary central nervous system lymphoma, Brain biopsy, Resection, Complications, Overall survival, Progression-free survival, prognosis

## Abstract

**Background:**

The aim of this study was to evaluate the effect of surgical resection and stereotactic biopsy on the complication rate, progression-free survival (PFS) and overall survival (OS) of 70 patients diagnosed at a single institution with primary central nervous system lymphoma (PCNSL) and to explore the predictors of selection for resection and the prognostic factors of PCNSL.

**Methods:**

A retrospective analysis was performed of 70 patients with PCNSL that was diagnosed by surgical resection or stereotactic brain biopsy in our department from January 2013 to May 2019. We divided the patients into two groups: a resection group (*n* = 28) and a stereotactic biopsy group (*n* = 42). Data on clinical characteristics, imaging findings, complication rates, PFS and OS were retrospectively reviewed and compared between these two groups. We also analysed the predictors of selection for resection and prognostic factors of PCNSL by multivariate analysis.

**Results:**

The median age was 53.3 ± 14.3 years, and there was a male predominance with a sex ratio of 1.33:1. The most common clinical manifestation was a headache. The complication rate in the resection group was 10.7% versus 7.1% in the stereotactic biopsy group, and there was no statistically significant difference. The rate of improvement in symptoms of the resection group was significantly higher than that of the stereotactic biopsy group. Multivariable analysis identified a single tumour and not involving deep structures as predictors of selection for resection. With a median follow-up of 30 months (range 1–110), the mean OS and PFS of all patients were 16.1 months and 6.2 months, respectively. Patients who underwent surgical resection had a mean OS of 23.4 months and PFS of 8.6 months versus 11.2 months and 4.6 months for those who had a brain biopsy performed. In addition, multivariable analysis showed that not involving deep structures and resection were favourable prognostic factors for PCNSL.

**Conclusions:**

The outcomes of patients with PCNSL treated in our cohort are still poor. In our series, surgical resection might play a role in significantly improving OS and PFS compared with stereotactic biopsy in a subset of patients. The type of surgery and tumour location are prognostic factors for PCNSL.

## Background

Primary central nervous system lymphoma (PCNSL) is a relatively rare and malignant brain tumour, most frequently presenting as diffuse large B-cell lymphoma (DLBCL), which is characterized pathologically by an angiocentric appearance, with lymphoma and inflammatory cells surrounding small blood vessels [1–3]. It is confined to the brain parenchyma, spinal cord, eyes, or leptomeninges but without involvement of systemic disease at the time the patients are diagnosed. Its overall incidence is 0.47 per 100,000 people per year in immunocompetent individuals, with a higher incidence in immunosuppressed patients [4]. The variables of clinical manifestations, imaging features and localization all contribute to the difficulty of making the diagnosis of PCNSL, which can easily be misdiagnosed as other malignancies, such as glioma and brain metastases. We usually perform stereotactic biopsy or craniotomy surgery to confirm the diagnosis. High-dose methotrexate chemotherapy has become the first-line treatment for PCNSL in the last 2 decades because it can cross the blood-brain barrier. Thus, the 5-year survival rate of PCNSL has increased but remains low at 33% [5, 6].

Currently, many scholars have studied the diagnostic procedure, treatment strategies and prognostic factors of PCNSL, but controversies still exist, and the optimal treatment of this disease remains a huge challenge due to the low incidence of PCNSL and the difficulty of conducting large clinical trials. In our study, we retrospectively reviewed patients treated for PCNSL in Tongji Hospital. The aim of the study was to identify the common clinical characteristics and to determine whether there were any differences in surgical outcomes and prognosis between stereotactic biopsied patients and patients who underwent resection.

## Materials and methods

### Patient selection and data collection

This retrospective study was permitted and sponsored by Tongji Hospital, Tongji Medical College, Huazhong University of Science and Technology. By searching the database, 70 patients with histological confirmation of PCNSL admitted to Tongji Hospital between January 1, 2013, and May 31, 2019, were identified. Their medical records were reviewed. Patients were divided into two groups according to the diagnostic procedure: a resection group and a stereotactic biopsy group. We assessed and reviewed the presenting symptoms and signs, Karnofsky performance status (KPS), International Extranodal Lymphoma Study Group (IELSG) score [2], imaging features, treatment outcomes and survival of all patients. Two neurosurgeons in our department independently performed and recorded detailed preoperative evaluations, including a careful history and a physical examination of all patients.

Patients underwent brain magnetic resonance imaging (MRI) on admission. The tumour location, involvement of brain structures, maximum diameter of the lesion, and the number of lesions were recorded. Postoperative complications were classified and recorded according to the Glioma Outcomes Project system, which involves systemic complications, regional complications, and neurologic complications [7].

Based on the experience of our single institution, we chose stereotactic biopsy in patients who were elderly, had a poor general condition, or seemed unlikely to tolerate the surgical resection procedure, or had multiple intracranial neoplasms or deep involved structures. Furthermore, we would consider surgical resection in patients with a good performance status and in a case of a single lesion where resection seemed safe. Additionally, when patients presented with signs of increased intracranial pressure and progressive neurological deficiencies, surgical resection was performed.

### Outcome and follow up

Patients were followed up after hospitalization and received MRI examinations during the follow-up period for the assessment of outcomes. Overall survival (OS) was calculated from the date of the diagnosis to the date of death or censoring. Progression-free survival (PFS) was calculated from the date of diagnosis to the date of disease progression.

### Statistical analysis

Statistical analysis was performed using SPSS Statistics 22.0 (IBM Corporation, Armonk, New York, USA). Clinical characteristics, imaging features and treatment outcomes were compared between the resection and stereotactic biopsy groups. For the description of continuous numerical variables, we used the mean ± standard deviation. The measurements and categorical data were statistically analysed with t-tests and χ2 tests, respectively. In addition, Fisher’s exact test and the Wilcoxon rank-sum test were used for between-group comparisons when appropriate. Probability values < 0.05 were considered to be statistically significant. We used the Kaplan-Meier method to estimate survival curves, and the log-rank test was used to compare different survival curves. Predictors of selection for resection were identified by using multiple-variable logistic regression. Multivariate analysis of variables with statistical significance in univariate analysis was performed by the Cox proportional hazards model.

## Results

### Patient characteristics

A total of 70 patients were identified, with 40 men (57.1%) and 30 women (42.8%). The mean age of the population was 53.3 ± 14.3 years, the mean KPS was 77.6 ± 15.8, and the mean duration of symptoms was 5.8 ± 11.2 months. There were no significant differences in patient age, sex, symptoms and signs or the duration of symptoms between the surgical resection group and the stereotactic biopsy group. The preoperative KPS of the surgical resection group was significantly higher than that of the stereotactic biopsy group (mean 84.3 ± 16.7 vs 73.1 ± 13.7, *P* = 0.002). Moreover, there was a significant difference in the IESLG score between the two groups (*P* = 0.006) (Table 1).

**Table 1:** Baseline clinical characteristics, treatment outcomes and prognosis for patients with PCNSL

Characteristics	Resection(*n* = 28)	Biopsy(*n* = 42)	Total(*n* = 70)	χ^2^/t	*P* value
Sex				0.972	0.324
Male	18	22	40		
Female	10	20	30		
Age	47.9 ± 15.9	56.9 ± 12.1	53.3 ± 14.3	2.686	0.995
KPS	84.3 ± 16.7	73.1 ± 13.7	77.6 ± 15.8	−3.068	0.002
IELSG score				10.303	0.006
Low risk(0–1)	16	9	25		
Intermediate risk(2–3)	10	22	32		
High risk(4–5)	2	11	13		
Present symptoms					
Headache	15	30	45	2.333	0.127
Limb weakness	12	22	34	0.610	0.435
Mental impairment	8	18	26	1.469	0.226
Aphasia	3	7	10	0.486	0.486
Ataxia	3	5	8	0.024	0.878
Seizure	2	3	5	0.000	1.000
Other	3	2	5	0.897	0.343
Duration of symptoms	4.3 ± 7.2	6.9 ± 13.3	5.8 ± 11.2	0.945	0.826
Numbers of tumors				14.593	0.000
Single	23	15	38		
Multiple	5	27	32		
The maximum diameter of the target	30.2 ± 10.6	29.7 ± 8.7	29.9 ± 9.5	−0.216	0.415
Location				22.451	0.000
Deep location	1	16	17		
Infratentorial	3	14	17		
Supratentorial	24	12	36		
Adjuvant therapies				1.324	0.516
Chemotherapy	14	18	32		
Radiotherapy	5	5	10		
Chemotherapy and radiotherapy	9	19	28		
Changes in symptoms				19.973	0.000
Aggravation	5	10	15		
Improvement	14	2	16		
Static	9	30	39		
Complication				0.273	0.601
No	25	39	64		
Yes	3	3	6		
Overall survival	23.4 ± 13.0	11.2 ± 6.2	16.1 ± 11.2	−5.263	0.000
Progression-free survival	8.6 ± 4.7	4.6 ± 3.2	6.2 ± 4.3	−4.241	0.000

### Tumour characteristics

Thirty-eight patients (54.3%) had a single lesion, and 32 patients (45.7%) had multiple lesions. In the resection group, 23 patients had a single lesion, and 5 patients had multiple lesions. In the stereotactic biopsy group, 15 patients had a single lesion, and 27 patients had multiple lesions. There was a significant difference in the numbers of lesions between the two groups. The mean size of all lesions was 29.9 mm: 30.2 mm in patients who underwent resection and 29.7 mm in patients who underwent stereotactic biopsy (*P* = 0.415) (Table 1).

### Adjuvant therapies

All patients were given chemotherapy and/or radiotherapy after resection or biopsy. Thirty-two patients (45.7%) received only high-dose methotrexate-based chemotherapy, 10 patients (14.3%) were treated with whole-brain radiation therapy alone, and 28 patients (40%) were given high-dose methotrexate-based chemotherapy with consolidation whole-brain radiation therapy. There was no significant difference in adjuvant therapies between the surgical resection group and the stereotactic biopsy group (*P* = 0.516) (Table 1).

### Treatment outcomes

Complications occurred in 3 cases in the resection group, including 1 patient with intracranial bleeding, 1 with cerebrospinal fluid leak and 1 with meningitis. In the biopsy group, 1 patient experienced intracranial bleeding, and 2 patients experienced seizures. Patients undergoing resection had comparable rates of complications as those undergoing stereotactic biopsy (10.7% vs 7.1%). However, the rate of improvement of symptoms of the resection group was significantly higher than that of the biopsy group (14/28 vs 2/42) (Table 1).

### Predictors of selection for resection

There was no difference favouring biopsy versus resection for sex, duration of symptoms or diameter of the tumours. On single-variable logistic regression, the following factors were significant: age, KPS, IESLG score, single tumour, and not involving deep structures. Multivariable analysis identified a single tumour and not involving deep structures as predictors of selection for resection (Table 2).

**Table 2:** Predictors of selection for resection

Independent variable	Single variable logistic regression			Multiple logistic regression		
	*p* value	OR	95%CI	*p* value	OR	95%CI
Sex	0.436	1.47	[0.56,3.84]	0.872	1.12	[0.28,4.58]
Age	0.016	0.95	[0.92,0.99]	0.607	0.99	[0.94,1.04]
KPS	0.005	1.05	[1.02,1.09]	0.122	1.04	[0.99,1.08]
IELSG score	0.009	9.778	[1.76,54.26]	0.462	0.08	[0.00,3.87]
Duration of symptoms	0.363	0.98	[0.92,1.03]	0.344	0.95	[0.86,1.06]
Numbers of tumors	0.000	0.12	[0.04,0.38]	0.016	0.17	[0.04,0.72]
The maximum diameter of the target	0.819	1.01	[0.96,1.06]	0.895	0.99	[0.92,1.07]
Location	0.000	6.91	[2.57,18.58]	0.001	6.18	[2.08,18.32]

### Prognosis

**Fig. 1:**
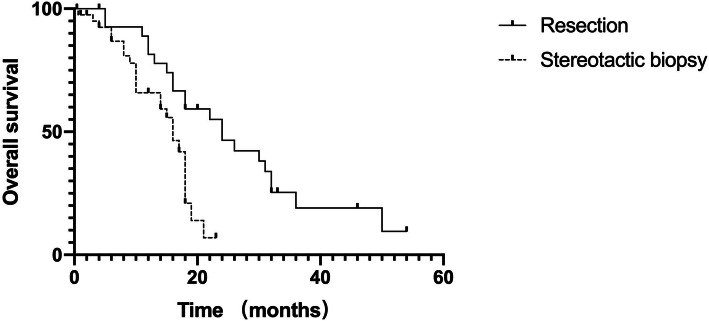
OS by type of surgery(*P* = 0.002)

**Fig. 2:**
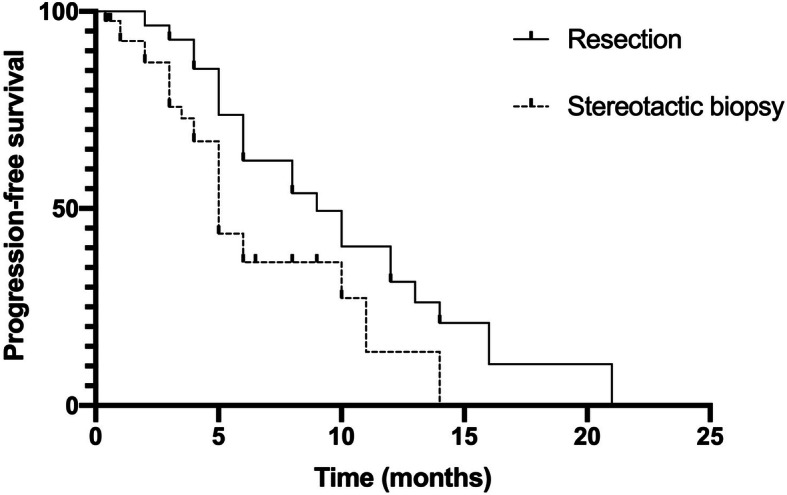
PFS by type of surgery(*P* = 0.038)

**Fig. 3:**
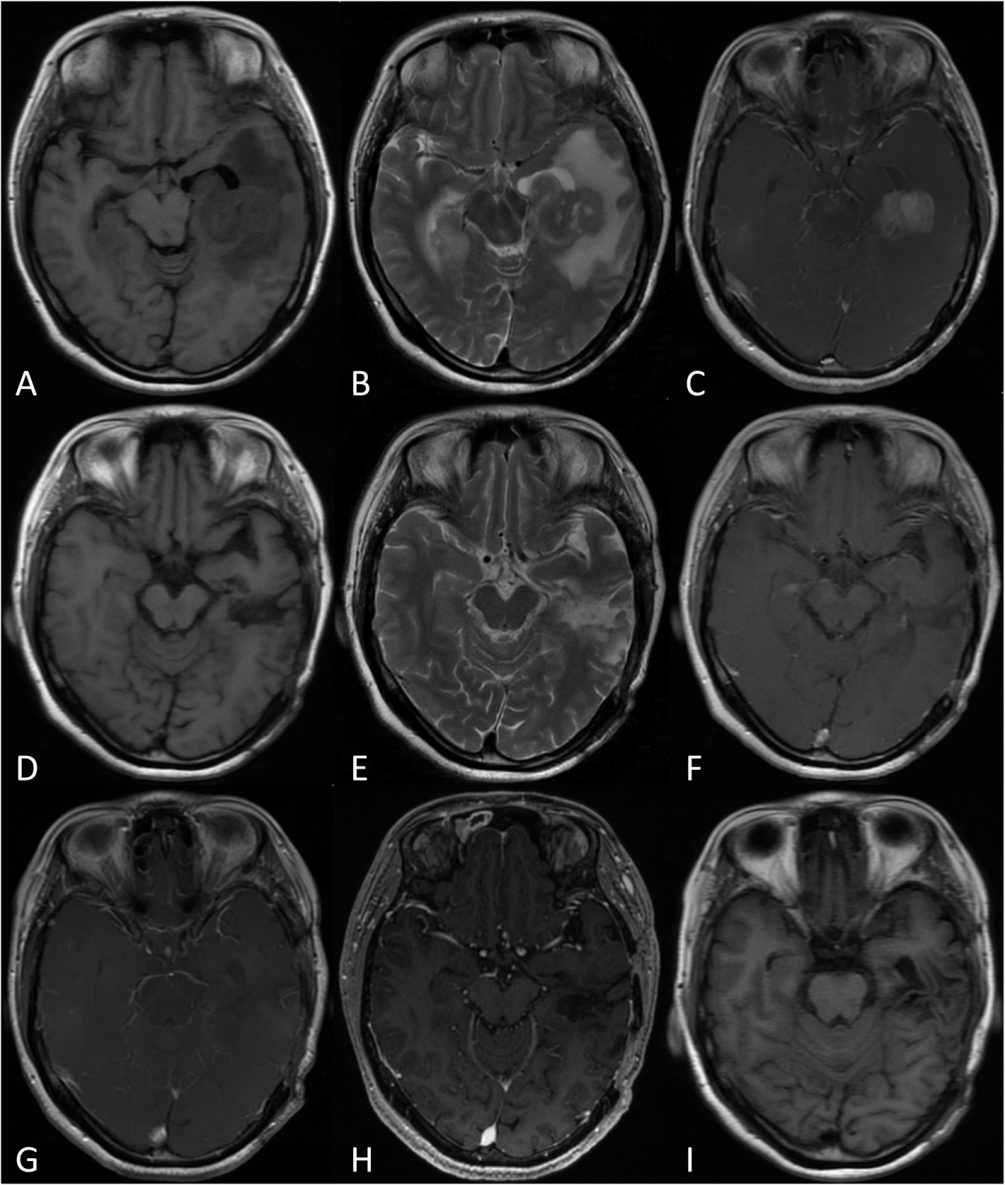
A 42-year-old men who presented with headache for 1 month. MRI (**a**, **b** and **c**) shows a contrast-enhancing solitary lesion with a diameter of 3.5 cm in the left temporal lobe. He underwent surgery with a craniotomy and gross total resection. The symptoms of headache were improved obviously. MRI examination (**d**, **e** and **f**) was taken at 1 week after surgery, which shows the lesion was removed completely. And MRI of 3 months (**g**), half a year (**h**) and 1 year (**i**) after surgery show that there was no recurrence of the tumor

With a median follow-up of 30 months (range 1–110), the mean OS and PFS of all patients were 16.1 months and 6.2 months, respectively. Patients who underwent surgical resection had a mean OS of 23.4 months and a PFS of 8.6 months versus 11.2 months and 4.6 months for those who had a brain biopsy performed (Table 1, Figs. 1 and 2). Table 3 summarizes the results from univariate analyses of OS and PFS. Younger age, lower IESLG score, not involving deep structures and resection were favourable prognostic factors for OS. In addition, deep structure, resection and no complications were identified as favourable prognostic factors for PFS. Sex, KPS, duration of symptoms, number of tumours, maximum diameter of the target, and changes in symptoms were not significant prognostic factors for survival (*P* > 0.05). After univariate analysis, multivariable Cox regression analysis was performed with the 5 statistically significant variables, which showed that deep structures not involved and resection were favourable prognostic factors for PCNSL (Table 4). There is a typical example of the resection group in Fig. 3.

**Table 3:** Univariate Analysis for Overall Survival and Progression-free Survival

	Number	Overall survival (months)	*P* value for OS	Progression-free survival (months)	*P* value for PFS
	70	16.1 ± 11.2		6.2 ± 4.3	
Sex			0.64		0.16
Female	34	15.6 ± 11.7		6.7 ± 4.6	
Male	36	16.6 ± 10.8		5.7 ± 3.9	
Age			0.02		0.28
< 60	44	18.7 ± 12.0		6.4 ± 4.6	
> =60	26	11.7 ± 8.1		5.8 ± 3.8	
KPS			0.10		0.66
> =70	51	16.1 ± 11.9		6.2 ± 4.3	
< 70	19	12.3 ± 8.0		6.7 ± 4.4	
IESLG score					
Low risk	25	23.0 ± 12.8	0.001	7.5 ± 5.2	0.138
Intermediate risk	32	13.6 ± 8.5		5.7 ± 3.4	
High risk	13	9.0 ± 6.1		4.8 ± 4.0	
Duration of symptoms (months)			0.21		0.22
> =1	53	16.7 ± 11.5		6.4 ± 4.6	
< 1	17	14.2 ± 10.1		5.5 ± 3.1	
Numbers of tumors			0.99		0.98
Multiple	32	10.6 ± 7.2		5.0 ± 4.0	
Single	38	20.8 ± 11.9		7.1 ± 4.3	
The maximum diameter of the target			0.25		0.10
> =30	38	16.9 ± 11.8		6.8 ± 4.8	
< 30	32	15.1 ± 10.5		5.5 ± 3.6	
Location			0.00		0.00
Not deep location	53	18.2 ± 11.7		6.9 ± 4.5	
Deep location	17	9.6 ± 6.1		3.8 ± 2.1	
The operative type			0.00		0.00
Biopsy	42	11.2 ± 6.2		4.6 ± 3.2	
Resection	28	23.4 ± 13.0		8.6 ± 4.7	
Changes in symptoms			0.94		0.96
Not improvement	54	14.9 ± 11.0		5.8 ± 4.0	
Improvement	16	20.0 ± 11.4		7.9 ± 4.9	
Complication			0.15		0.00
No	58	16.7 ± 10.1		6.9 ± 4.3	
Yes	12	13.1 ± 15.8		2.8 ± 1.9

**Table 4:** Multivariate analysis for OS and PFS

	*P* value
Age	NS
IESLG score	NS
Deep location	0.00
The type of surgery	0.00
Complication	NS

## Discussion

PCNSL is defined as an extranodal non-Hodgkin’s lymphoma, accounting for 2–5% of all primary central nervous system neoplasms, with a more aggressive course and a poor prognosis compared with other lymphomas [6, 8, 9]. The clinical characteristics of our patients were consistent with the literature. Their median age was 53.3 years, and there was a male predominance with a sex ratio of 1.33:1, which has been previously seen in many previous large series [2, 3, 8, 10]. PCNSL mostly presents with neurologic impairment symptoms as the first manifestation rather than common B symptoms (fever, weight loss, night sweats). Patients presented with various symptoms based on the CNS site involved, the most common being headache (64.3%) in our cohort, followed by limb weakness and mental impairment, less frequently presenting with aphasia, ataxia, seizure and other symptoms. Additionally, seizures are relatively uncommon because PCNSL tends to involve deep brain structures. The mean duration of symptoms to diagnosis was 5.8 months in our study.

PCNSL has typical radiological appearances, which often show diffuse homogeneous contrast enhancement and surrounding vasogenic oedema on MRI (Fig. 3). There are also many atypical imaging features that may mimic other diseases and add to the diagnostic difficulty. Recently, various advanced imaging techniques have also been used in the diagnosis and prognostication of PCNSL, including diffusion tensor imaging (DTI), diffusion-weighted imaging (DWI) and magnetic resonance spectroscopy (MRS). Toh et al. [11] found that the fractional anisotropy of DTI is significantly lower in PCNSL than in glioblastoma. In addition, PCNSL has markedly higher choline/creatine and choline/N-acetyl aspartate ratios on MRS than other glial tumours [12]. In our present study, we also observed that the most common location of PCNSL was the supratentorial location (51.4%), followed by the infratentorial and deep locations (24.3%). This is consistent with a retrospective analysis of the clinical data of 100 patients with PCNSL, which was reviewed and analysed by Küker et al [13] At the same time, we found that 65% of PCNSL patients had solitary lesions, and 35% had multiple lesions. The mean maximum diameter of the tumour was 29.9 mm.

All of these clinical characteristics enable us to better differentiate PCNSL from other diseases, including gliomas, metastasis, infections and inflammatory demyelinating disease. However, to create favourable conditions for comprehensive follow-up therapy, biopsy or resection must be performed to obtain histopathologic or cytologic confirmation of the diagnosis. A series of studies have been carried out to determine optimal treatment regimens to obtain a better survival benefit for PCNSL patients [3, 10, 14, 15], but there is still controversy. Jahr et al. [16] reported that resection surgery played no role in significantly improving either OS or PFS in PCNSL patients. However, Bellinzona et al. [15] demonstrated that surgery might have a role in a selected subset of patients presenting with large single space occupying lesions and deteriorating neurological status. Cloney et al. [17] suggested that the overall complication rate of resection for PCNSL is comparable to rates for other CNS malignancies and believed that resection is safe for selected patients. In our series of 70 patients, 42 underwent stereotactic biopsy, and 28 underwent surgical resection. The complication rate was 7.1% for stereotactic biopsy and 10.7% for resection, and there was no significant difference. Resection for PCNSL patients has been discouraged and traditionally limited due to an unacceptable morbidity for the procedure according to the literature from the 1970s to 1990s. However, with the development of modern surgical techniques, especially neuronavigation, fluorescein for tumour visualization and intraoperative neurophysiologic monitoring, which have improved the safety and accuracy of surgery, the postsurgical complication rates for PCNSL have decreased to 0–20%, as the recent literature has reported [10, 14, 17]. For example, Cloney et al. [17] reported that the complication rate of the resection group (17.2%) was lower than that of the biopsy group (28.2%) through the analysis of 129 patients with PCNSL between 2000 and 2015, and they deemed resection safe for selected patients. Our analysis revealed no significant difference in the complication rate following resection compared to stereotactic biopsy. Our complication rate for resection demonstrates a decrease in comparison with the previously published literature, which was 40% in PCNSL patients undergoing resection. We also observed that the symptom improvement rate of the resection group was 50%, which was significantly higher than that of the biopsy group (4.8%). Furthermore, when patients present with signs of increased intracranial pressure and progressive neurological deficits, they would benefit more from surgery than stereotactic biopsy.

In our follow-up study, the mean overall survival was 16.1 months, and the PFS was 6.2 months. In addition, patients who underwent resection had a mean OS of 23.4 months and PFS of 8.4 months, 11.2 months and 4.6 months for those who had a biopsy performed. Weller et al. [18] found that OS and PFS were significantly longer in the resection subset than in biopsied patients through a large randomized phase III study comprising 526 patients. Jahr et al. [16] also reported that patients who underwent resection had an insignificant prolongation of OS compared with patients who had a biopsy performed. In addition, Rae et al. [19] performed a study that involved the largest collective sample of 13,704 patients and showed an increased OS benefit with resection compared with stereotactic biopsy. Our results obtained in both groups are in accordance with those reported in the literature. We speculate that this may be because of the fractional cell killing of cytotoxic drugs, that is, the fewer tumour cells that are present at the start of chemotherapy, the fewer cycles are needed to induce a complete remission. Another reason may be that patients who underwent resection had a reduced likelihood of chemotherapy-induced mutations, which reduced their risk of developing chemotherapy resistance. The prognosis of PCNSL is still poor, and multivariate analysis of various patient characteristics available for most patients identified tumour location and the type of surgery as the only significant prognostic factors for both PFS and OS.

In our study, we also analysed patient characteristics when making management decisions for resection or biopsy. We found that age, KPS, IESLG score, a single lesion, and a not deep lesion location were factors associated with selection for resection on single variable logistic regression. However, in multivariable analysis, we identified that a single tumour and not deep structures were predictors of selection for resection. Our findings are consistent with Cloney et al.’s series of 129 patients with PCNSL [17], which also found that age, multiple lesions and deep lesions influenced selection for resection. Weller et al. [18] also found that patients with a single lesion were likely to undergo resection in their PCNSL series. The guidelines from the European Association only recommend resection when there is a mass effect causing herniation [20]. However, Schellekes et al. [21] found that specific subgroups of patients with a solitary PCNSL lesion might gain a survival benefit from resection compared with undergoing only a diagnostic biopsy. Additionally, in light of our findings and considering that surgical resection might benefit additional patients, not just patients with signs of increased intracranial pressure and progressive neurological deficits, because it can help patients to relieve symptoms quickly, such as headache, limb weakness, vomiting, etc., which in turn can improve their quality of life and their tolerance of upcoming intensive chemotherapy and radiation treatment. In brief, resection surgery might be better for a selected subset of PCNSL patients, with better OS and PFS and complication rates comparable to the rates of the biopsy group. When making management decisions for PCNSL patients, we should take individual patient characteristics into account.

There are several limitations of our study. The primary limitations are its retrospective design and the limited number of patients. Moreover, the findings are clearly limited by the presence of selection bias, as described previously, and the lack of multicentre participation. Because of a relatively small analysed study population of our single-center, data should be cautiously used. However, we found that surgery might play a positive role in a subset of patients, which suggest the need for further prospective randomized studies to better evaluate the efficacy and safety of surgical resection for PCNSL patients.

## Conclusion

The outcomes of patients with PCNSL treated in our cohort are still poor. In our series, surgical resection might play a role in significantly improving OS and PFS compared with stereotactic biopsy in a subset of patients. The type of surgery and tumor location are the prognostic factors of PCNSL.

## Data Availability

The datasets used and/or analysed during the current study are available from the corresponding author on reasonable request.

## Abbreviations

PFS: Progression-free survival
OS: Overall survival
PCNSL: Primary central nervous system lymphoma
DLBCL: Diffuse large B-cell lymphoma
KPS: Karnofsky performance status
IELSG: International Extranodal Lymphoma Study Group
MRI: Magnetic resonance imaging
DTI: Diffusion tensor imaging
DWI: Diffusion-weighted imaging
MRS: Magnetic resonance spectroscopy
